# Osthole Inhibits Expression of Genes Associated with Toll-like Receptor 2 Signaling Pathway in an Organotypic 3D Skin Model of Human Epidermis with Atopic Dermatitis

**DOI:** 10.3390/cells11010088

**Published:** 2021-12-28

**Authors:** Natalia Karolina Kordulewska, Justyna Topa, Robert Stryiński, Beata Jarmołowska

**Affiliations:** 1 Department of Biochemistry, Faculty of Biology and Biotechnology, University of Warmia and Mazury in Olsztyn, 10-719 Olsztyn, Poland; robert.stryinski@uwm.edu.pl (R.S.); bj58@wp.pl (B.J.); 2 Laboratory of Translational Oncology, Intercollegiate Faculty of Biotechnology, Medical University of Gdańsk, 80-211 Gdańsk, Poland; justyna.topa@gumed.edu.pl

**Keywords:** fexofenadine, clobetasol propionate, lipopolysaccharide, nuclear factor kappa B, TIR domain-containing adaptor protein, interleukin receptor-associated kinase

## Abstract

The Toll-like receptor (TLR) family signature has been linked to the etiopathology of atopic dermatitis (AD), a chronic inflammatory skin disease associated with skin barrier dysfunction and immune system imbalance. We aimed to investigate whether osthole (a plant-derived compound) can inhibit the genetic profile of key genes associated with TLR2 signaling (*TIRAP*, *MyD88*, *IRAK1*, *TRAF6*, *IκBα*, *NFκB*) after stimulation with LPS or histamine in a 3D in vitro model of AD. Overexpression of the aforementioned genes may directly increase the secretion of proinflammatory cytokines (CKs) and chemokines (ChKs), which may exacerbate the symptoms of AD. Relative gene expressions were quantified by qPCR and secretion of CKs and ChKs was evaluated by ELISA assay. LPS and histamine increased the relative expression of genes related to the TLR2 pathway, and osthole successfully reduced it. In summary, our results show that osthole inhibits the expression of genes associated with the TLR signaling pathway in a skin model of AD. Moreover, the secretion of CKs and ChKs after treatment of AD with osthole in a 3D skin model in vitro suggests the potential of osthole as a novel compound for the treatment of AD.

## 1. Introduction

Atopic dermatitis (AD) is a chronic inflammatory skin disease associated with skin barrier dysfunction and immune system imbalance. AD is a global public health problem due to its increasing prevalence and socioeconomic burden [1]. Approximately 20% of people worldwide suffer from AD. The disease is diagnosed in children and adults, the latter being the most commonly affected (36.8% of cases) [2] and almost half of them suffer from the persistent and chronic pattern of AD [3]. This skin disease is characterized by clinical manifestations of dry skin, chronic eczema and severe itching, and often occurs in combination with asthma, allergic rhinitis and genetic predisposition [4]. The literature indicate that AD patients have (i) elevated blood eosinophil and serum IgE levels (mainly in extrinsic, not intrinsic AD), (ii) elevated levels of serum inflammatory cytokines (CKs) and chemokines (ChKs), such as IL-1β, IL-4, IL-6, IL-8 and tumor necrosis factor alpha (TNF-α), and (iii) infiltration of Th2 cells, mast cells and eosinophils in the skin [5,6,7].

Treatment of AD usually relies on the administration of drugs, including antihistamines and glucocorticoids, but more advanced therapies such as immunotherapy have also been used. However, these therapies are not commonly used due to side effects [8]. Several new drugs have been developed, including monoclonal antibodies (dupilumab) and new topical molecules (tofacitinib, crisaborol) designed to block the specific mechanism of AD. These new therapeutics have heralded a new era in the treatment of AD, but the results of clinical trials did not show satisfactory efficacy [9]. Therefore, recent studies have focused on alternative drugs to control AD [10].

The literature indicates that Toll-like receptor 2 (TLR2) and TLR2 gene polymorphisms play an important role in the development of AD [11,12]. In addition, TLR2/4 was found to be downregulated in peripheral blood mononuclear cells (PBMCs) isolated from AD patients [13,14]. TLR2 is expressed in cells of the innate immune system, such as dendritic cells and macrophages, but also in non-immune cells, such as fibroblasts and epithelial cells.

**Figure 1:**
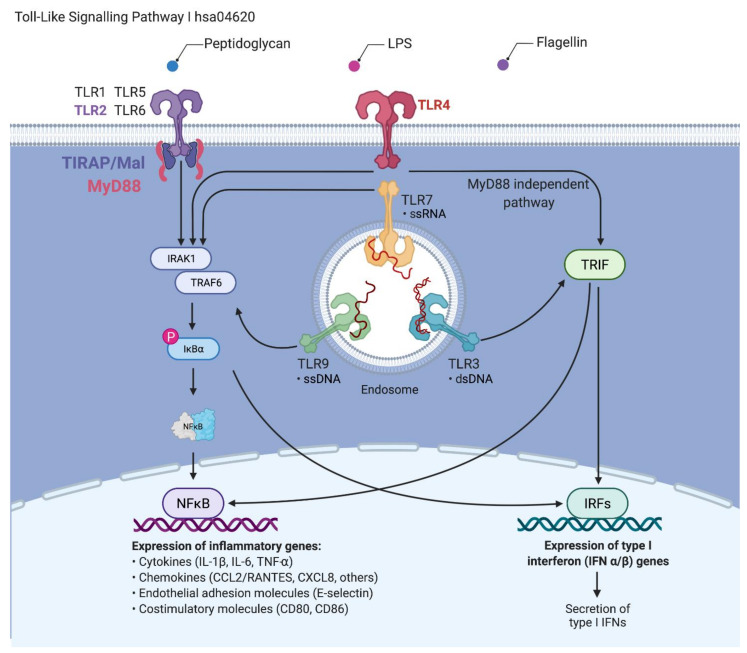
The signaling pathway of Toll-like receptors (TLRs). Upon contact with pathogens that secrete pathogen-associated molecular patterns, their recognition is initiated *via* pattern recognition receptors, including TLRs. These receptors trigger innate immune responses *via* the MyD88-dependent pathway, leading to the production of proinflammatory cytokines with activation of nuclear factor kappa B (NFκB) and downstream gene targets [37,38]. The MyD88-independent pathway associated with the induction of type I interferon (IFN) and IFN-inducible genes. (Data source: KEGG pathway, hsa04620 and compiled in Biorender.com (Accessed on 18 December 2021). Adapted from “TLR Signaling Pathway”, by BioRender.com (2021). Retrieved from https://app.biorender.com/biorender-templates).

Our analyses of differentially expressed genes in the model of AD after stimulation by histamine or lipopolysaccharide (LPS) identified the TLR2 and nuclear factor kappa B (NFκB) as the most deregulated in the 3D model of AD. Activation of TLRs (Figure 1) recruits several downstream adaptation molecules, including myeloid differentiation protein 88 (MyD88) which contributes to signal amplification by interleukin receptor-associated kinase 1 (IRAK1). Convergence of IRAK1 with NFκB induces transcription of IL-6, which counteracts invader threat and is involved in stimulation of other immune components [15,16,17]. Identification of the molecular profile of these genetic signatures could provide new diagnostic and clinical treatment options in AD. In this sense, the present work aimed to assess the genetic profile of key players associated with the TLR pathway, i.e., TIR domain-containing adaptor protein (TIRAP), MyD88, IRAK1, TNF receptor-associated factor 6 (TRAF6), NFκB inhibitor alpha (IκBα) and NFκB in a stimulated model of AD and after treatment with osthole, in order to evaluate the changes in gene expression signature and secretion of CKs and ChKs.

Osthole (7-methoxy-8-(3-methylbut-2-en-1-yl)-2*H*-chromen-2-one), a bioactive coumarin derivative derived from many medicinal plants, is widely used in herbal medicine and functional foods. Osthole is extracted from the ripe fruits of *Cnidium monnieri* and plants belonging to the genera *Angelica*, *Archangelica*, *Citrus* and *Clausena*. The fruits of *C. monnieri* are widely used in traditional Chinese medicine to strengthen the immune system, relieve rheumatic pain and treat asthma, osteoporosis and skin diseases [18]. This natural coumarin has potent immunomodulatory effects on innate and adaptive immunity [19]. In-depth studies have shown that osthole has a wide range of different pharmacological effects, including antiallergic [20,21,22,23,24,25,26], anti-inflammatory [27], antioxidant [28], hepatoprotective [29], neuroprotective [30], antiosteoporotic [31] and antimicrobial properties [32].

Literature data clearly indicate the involvement of pro-inflammatory factors (such as thymic stromal lymphopoietin, IL-33, IL-25) in the development of AD upon contact with allergens, antigens, viruses, bacteria and fungi. Therefore, we used histamine as an allergic activator and LPS as a bacterial trigger to induce inflammation in a 3D skin model [33,34,35,36]. Then, we examined how osthole alleviates the effects of the inflammatory triggers and compared its effect with substances that served as positive controls—clobetasol propionate (CP), a corticosteroid used to treat various skin diseases, and fexofenadine (FXF), a third-generation antihistamine.

We hypothesized that: (i) osthole inhibits the secretion of IL-1β, IL-6, IL-8, TNF-α, chemokine (C-C motif) ligand 2/monocyte chemoattractant protein 1 (CCL2/MCP-1), chemokine (C-C motif) ligand 5/regulated on activation, normal T cell-expressed and secreted (CCL5/RANTES) and cyclooxygenase 2 (COX-2), which are increased after histamine and LPS treatment, (ii) osthole inhibits the expression of *COX-*2 and key TLR2 pathway genes (*TIRAP*, *MyD88*, *IRAK1*, *TRAF6*, *IκBα* and *NFκB*) in a 3D organotypic skin model; (iii) osthole can be considered as an herbal agent for the treatment of inflammatory skin diseases.

## 2. Materials and Methods

### 2.1. Chemicals

Histamine (CAS 51-45-6), LPS from *Escherichia coli* O111:B4 (LPS, EC 297-473-0), FXF (CAS 153439-40-8), osthole (CAS 484-12-8), and CP (CAS 25122-46-7) were obtained from Sigma Aldrich (St. Louis, MO, USA, cat. no. Y0001779, L4391, Y0000789, Y0001207 and Y0000559, respectively). Histamine and LPS were dissolved in water, FXF and CP were dissolved in dimethyl sulfoxide (DMSO, Sigma Aldrich, St. Louis, MO, USA, cat. no. D8418) and osthole was dissolved in 96% ethanol (Chempur, Piekary Śląskie, Poland, cat. no. 653964200). The solutions were filtered through 0.22 µm pore filters, aliquoted and stored at −20 °C.

### 2.2. Cell Culture

Normal Human Epidermal Keratinocytes (NHEK; juvenile foreskin, pooled) were purchased from PromoCell GmbH (Heidelberg, Germany, cat. no. C-12005) and grown in T-75 flasks in keratinocyte medium (Keratinocyte Growth Medium 2, PromoCell, Heidelberg, Germany, cat. no. C-20011). The medium and supplements were mixed according to the manufacturer’s instructions. The NHEKs were incubated at 37 °C in a 95% humidified atmosphere and 5% CO_2_. The culture medium was changed every 2–3 days, and cells were passaged when confluency reached approximately 70–90%. Only early passages (3–7) of keratinocytes were used in this study.

Normal Human Dermal Fibroblasts (NHDF; from juvenile foreskin) were purchased from PromoCell GmbH (Heidelberg, Germany, cat. no. C-12300) and cultured in a T-75 flask in recommended culture medium (PromoCell GmbH, Heidelberg, Germany, cat. no. C-23010). The medium and supplements were mixed according to the manufacturer’s instructions and contained 1% penicillin/streptomycin (Sigma Aldrich, St. Louis, MO, USA, cat. no. P4333). The NHDF cell line was incubated at 37 °C in a 95% humidified atmosphere and 5% CO_2_. The culture medium was changed every 2–3 days and cells were passaged when confluence reached approximately 80–90%.

### 2.3. Organotypic 3D Skin Model

**Figure 2:**
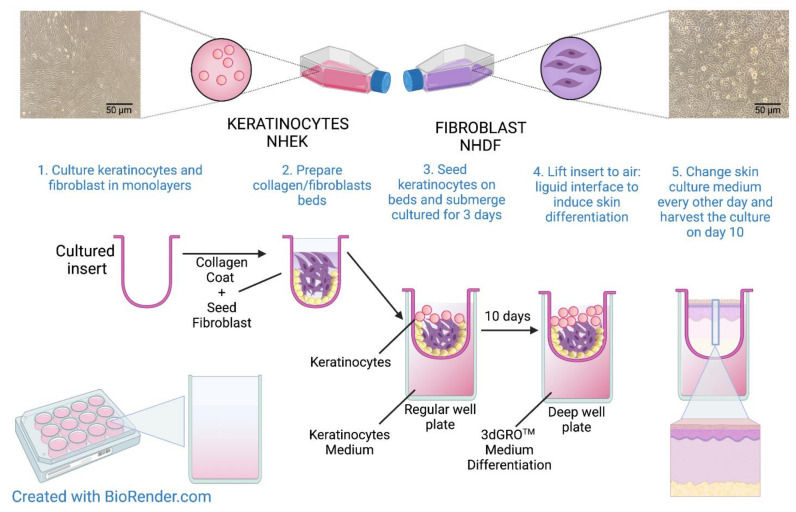
Overview of the preparation of organotypic 3D human skin model. Created using BioRender.com.

**Figure 3:**
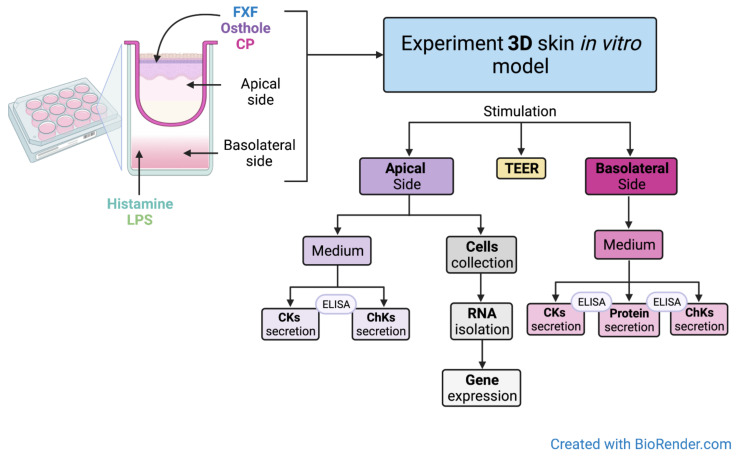
Experimental setup. Created with BioRender.com.

Since the viability and undifferentiation of the NHEK and NHDF cell lines are crucial for the construction of the 3D skin model, only cells to passage 5 were used in the experiment. The 3D skin model was constructed on the collagen/fibroblast bed, which was prepared as follows: 6 mL of rat tail collagen (Sigma Aldrich, St. Louis, MO, USA, cat. no. 08-115) was added to a 15 mL conical tube, then 1.6 mL of reconstitution buffer (1.1% NaHCO_3_, 0.025 N NaOH, 100 mM HEPES, 5× DMEM/F12) were added and mixed gently to prevent bubble formation. Then, 1.6 × 10^6^ NHDFs suspended in 400 µL of medium (4 × 10^6^ cells/mL) were added to the reconstituted collagen and mixed. Next, 400 µL of the NHDFs/collagen matrix mixture was added directly to the center of each MilliCell 0.4 μm pore insert (Millipore, Burlington, MA, USA, cat. no. MCHT12H48) in a 12-well plate (Greiner Bio-One, Kremsmünster, Austria, cat. no. 665110) and incubated at 37 °C for at least 30 min to allow for complete polymerization. After that, 2 × 10^5^ NHEKs suspended in 0.5 mL of medium (4 × 10^5^ cells/mL) were added to each collagen/fibroblast bed containing NHDF cells in polymerized collagen. Then, 2 mL of keratinocyte medium was added to the outside of the inserts. The cells were incubated overnight at 37 °C. The next day, another 0.5 mL of keratinocyte medium were added to the inside of each insert and incubated at 37 °C for 2 more days. The NHEKs were maintained in submerged culture on the collagen beds for a total of three days. On day 4, the medium was carefully aspirated from each insert. Next, the inserts were transferred to a new 12-well plate containing 4.5 mL of 3dGRO™ Skin Differentiation Medium (Sigma Aldrich, St. Louis, MO, USA, cat. no. SCM310) in each well. The skin culture was incubated at 37 °C for another 10 days. After that time, approximately 8 to 10 layers of differentiated epithelium had formed. An overview of the organotypic human skin culture protocol is described in Figure 2. The experimental setup with detailed graphical representations of the performed analyses is shown in Figure 3.

### 2.4. Incubation of the Organotypic 3D Skin Model with the Investigated Substances

The culture inserts were transferred to new deep-well plates. The medium containing 2 µg/mL LPS, or 100 µg/mL histamine was added to the basolateral side and after 3 h of incubation, different concentrations of FXF, CP (0.125, 0.5 mg/mL) and osthole (0.0625, 0.125, 0.25, 0.5 mg/mL) were applied to the apical side of the insert. FXF, osthole, and CP were also added to the insert without prior stimulation with histamine or LPS. After 72 h of incubation, the media (from the apical and basoteral sides) were collected for analysis of CKs levels, and skin cells were collected from the insert for total RNA isolation and reverse transcription.

### 2.5. RNA Isolation and Reverse Transcription

Total RNA was isolated according to the protocol described by Kordulewska et al. [39]. In brief, extraction of total RNA from 3D skin cells was performed using a TRIzol™ reagent (Invitrogen, Thermo Fisher Scientific, Waltham, MA, USA, cat. no. 15596026) according to the manufacturer’s protocol. RNA concentration and purity were determined using NanoDrop ND-1000 spectrophotometer (NanoDrop Tech., Inc. Wilmington, DE, USA). The High-Capacity cDNA Reverse Transcription Kit (Applied Biosystems, Thermo Fisher Scientific, Waltham, MA, USA, cat. no.4368814) was used for reverse transcription according to the manufacturer’s protocol.

### 2.6. Quantitative Real-Time PCR (qPCR) and Data Analysis

Gene expression was analyzed by quantitative real-time polymerase chain reaction (qPCR). The expression of *COX-*2, *TLR2*, *TIRAP*, *MyD88*, *IRAK1*, *TRAF6*, *IκBα*, *NFκB* and tyrosine-3-monooxygenase (*YWHAZ*) was examined. *YWHAZ* was used as a reference gene to normalize disproportion in mRNA amount. The validated primers are listed in Table S1. The qPCR was performed with the QuantStudio™ 3 Real-Time PCR System (Applied Biosystems, Foster City, CA, USA) using the FastStart Essential DNA Green Master Kit (Roche Diagnostics, Basel, Switzerland, cat. no. 06402712001). Additionally, 5 ng of cDNA was used per reaction and qPCR was performed in triplicate under the following conditions: denaturation at 95 °C for 10 min, amplification and quantification repeated 45 times (95 °C for 20 s, 60 °C for 20 s and 72 °C for 20 s with a single fluorescence measurement), melting curve at 60–95 °C with a heating rate of 0.1 °C per second and continuous fluorescence measurement and final cooling to 4 °C. Gene expression was analyzed according to Pfaffl [40]. Results were scaled to the expression level of the control determined as one.

### 2.7. Analysis of Cytokine and Chemokine Levels

Levels of IL-1β, IL-6, IL-8, and TNF-α were determined using enzyme-linked immunosorbent assay (ELISA) kits from Diaclone (Besancon, France; IL-1β—cat. no. 851.610.001, TNF-α—cat. no. 851.570.001), Mabtech (Nacka Strand, Sweden; IL-6—cat. no. 3460-1H-20) and BD Biosciences (San Jose, CA, USA; IL-8—cat. no. 555244). Kits for analysis of CCL5/RANTES, CCL2/MCP-1 and COX-2 levels were from Abcam (Cambridge, UK; Human RANTES ELISA Kit, cat. no. ab100633, Human MCP-1 ELISA Kit, cat. no. ab179886, and Human COX-2 ELISA Kit, cat. no. ab267646). Samples were tested in quadruplicate. Results were standardized by comparison to a standard curve.

### 2.8. Measurement of Transepithelial Electrical Resistance

To assess the integrity of tight junctions in the organotypic 3D skin model in the presence of the tested compounds, transepithelial electrical resistance (TEER) was measured using a Millicell ERS-2 volt-ohm meter (Merck, St. Louis, MO, USA, cat. no. MERS00002). The measurement of TEER was performed in a prepared 3D skin model that reached an appropriate TEER value (at least 250 Ω × cm^2^) immediately after the medium was replaced with a keratinocyte growth medium containing LPS, histamine, FXF, osthole, CP and their mixtures (time 0) and after 1, 2, 3, 6, 24 and 48 h of incubation with the tested substances. The TEER value was calculated according to the formula described in Srinivasan et al. [41]. Because the TEER values were initially different between wells, measurements at different time points were expressed as a percentage of the TEER value at time 0 (100%).

### 2.9. Statistical Analysis

Data analysis and visualization were performed using GraphPad Prism software version 9.3.1 (GraphPad Software, San Diego, CA, USA) and presented as mean ± standard error of the mean. Ordinary one-way ANOVA with Tukey’s multiple comparison test and ordinary two-way ANOVA with Dunnett’s multiple comparison test were used to examine differences between quantitative values. The statistical significance level was set at a *p*-value < 0.05.

## 3. Results

### 3.1. Osthole Prevents Histamine- and LPS-Induced Disruption of Tight Junctions in a 3D Organotypic Skin Model

**Figure 4:**
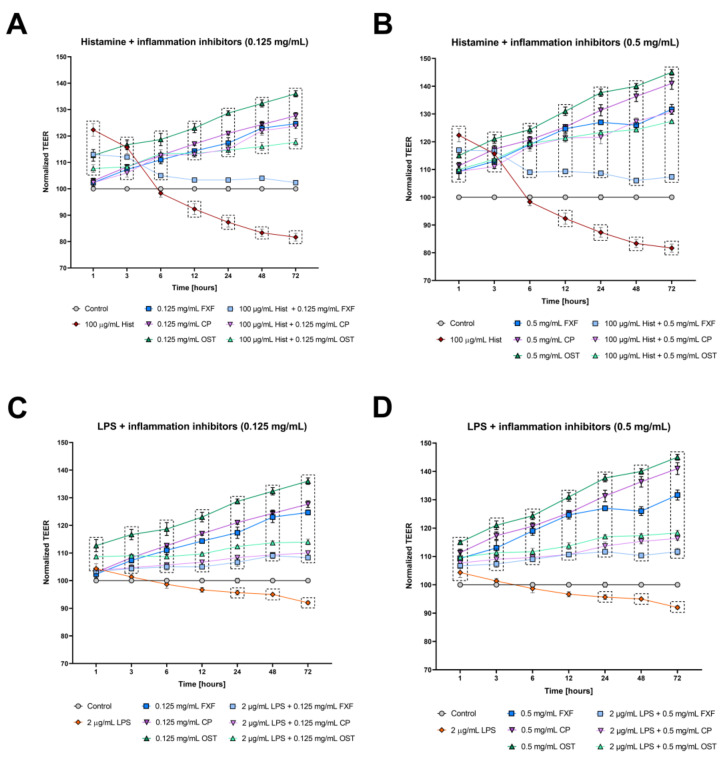
Changes in integrity of cultured cells monolayer measured by TEER in the organotypic 3D skin model after incubation with histamine (Hist; **A**,**B**) lipopolysaccharide (LPS; **C**,**D**), fexofenadine (FXF), clobetasol propionate (CP) and osthole (OST), and mixtures of histamine and LPS with FXF, CP and osthole. Symbols indicate mean and bars indicate standard error of the mean. Statistically significant differences (*p* < 0.05, two-way ANOVA with Dunnett’s multiple comparisons test) are shown in rectangles with dotted edges.

The integrity of tight junctions in the organotypic 3D skin model was determined by measuring TEER to assess the effects of osthole on barrier function. Stimulation with histamine resulted in a significant increase in TEER at the beginning of experiment (1 and 3 h), reaching the level of control after 6 h of incubation (Figure 4A,B), but from 12 h of experimentation, the levels of TEER were significantly lower compared with the control. Osthole significantly improved the integrity of the 3D skin model after treatment with histamine, and its effect was like that of CP, indicating its anti-inflammatory properties (Figure 4A,B). FXF at a concentration of 0.125 mg/mL significantly increased TEER levels at 1, 3 and 6 h of the experiment, but from 12 h of the experiment onward, it had no effect on permeability (Figure 4A). On the other hand, FXF at a higher concentration (0.5 mg/mL) increased the levels of TEER throughout the experiment, but its effect was weaker compared with osthole and CP (Figure 4B). After stimulation with LPS, TEER levels increased at the first time point. After 3 to 24 h, the TEER values reached the level of the control, and from 48 h of the experiment, the TEER values were significantly lower compared with the control (Figure 4C,D). Osthole significantly improved 3D skin integrity after LPS treatment, at similar levels to CP and FXF (Figure 4C,D). FXF, CP and osthole alone significantly improved cells integrity, with the latter causing the highest increase in TEER value (Figure 4).

### 3.2. Osthole Decreases Secretion of Pro-Inflammatory Cytokines, Chemokines and Proteins in a 3D Organotypic Skin Model Treated with Histamine and LPS

**Figure 5:**
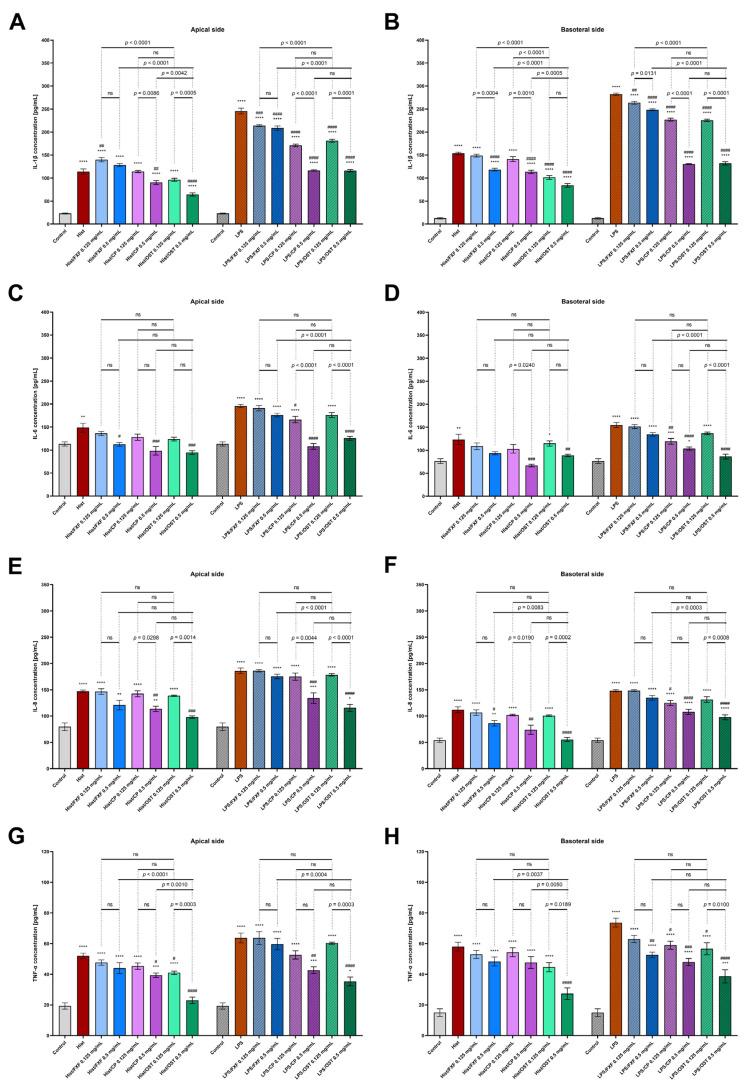
Secretion of IL-1β (**A**,**B**), IL-6 (**C,D**), IL-8 (**E**,**F**) and TNF-α (**G**,**H**) in 3D skin model after incubation with histamine (Hist; 100 µg/mL) and LPS (2 µg/mL) alone and in the mixtures with fexofenadine (FXF), clobetasol propionate (CP) and osthole (OST; 0.125 and 0.5 mg/mL) on the apical (**A**) and basolateral sides (**B**). The horizontal line shows the mean and the bars show the standard error of the mean. Statistically significant differences (one-way ANOVA with Tukey’s multiple comparisons test) compared to control (*—*p* < 0.05, **—*p* < 0.01, ***—*p* < 0.001, ****—*p* < 0.0001) and to cells treated with histamine or LPS (#—*p* < 0.05, ##—*p* < 0.01, ###—*p* < 0.001, ####—*p* < 0.0001) are marked; ns—non-significant.

To determine whether histamine and LPS trigger inflammation and osthole reduces this effect, secretion of pro-inflammatory CKs (IL-1β, IL-6, and TNF-α), ChKs (IL-8, CCL5/RANTES and CCL2/MCP-1) and proteins (COX-2) was examined in a 3D organotypic skin model. Inflammatory mediators significantly increased IL-1β levels on both the apical (Figure 5A) and basolateral sides (Figure 5B). Osthole successively decreased histamine- and LPS-induced IL-1β secretion in a concentration-dependent manner. The lowest concentration of IL-1β was measured after stimulation with osthole at the dose of 0.5 mg/mL, which was significantly lower than when cells were treated with the same concentration of FXF and CP (only in histamine-induced cells). The obtained results clearly indicate the blocking of IL-1β secretion induced by inflammatory mediators. Importantly, osthole not only prevented IL-1β secretion under inflammatory conditions (histamine or LPS/osthole treatment), but also decreased IL-1β secretion alone in a concentration-dependent manner (Figure S1C). The same trend was observed after incubation with CP (Figure S1B), and the least IL-1β inhibition was observed after incubation with FXF (Figure S1A).

Histamine and LPS significantly increased IL-6 secretion into the culture medium at the apical (Figure 5A,C) and basoteral sides (Figure 5D) of the inserts. The highest concentration of osthole inhibited the secretion of IL-6 comparable to the level of control. There was no significant difference between the secretion of IL-6 by cells stimulated with osthole and CP, confirming that osthole has comparabl e anti-inflammatory properties to this glucocorticosteroid. Moreover, osthole not only inhibited the secretion of IL-6 under inflammatory conditions (stimulation with histamine or LPS), but also decreased the secretion of IL-6 when cells were incubated with osthole alone (Figure S1F). Similar changes were observed for CP, but the statistical significance was lower (Figure S1E).

Osthole significantly reduced the secretion of IL-8 in the 3D skin model incubated with histamine and LPS (Figure 5E,F), and its effect was comparable to that of CP, as no significant differences were observed. Moreover, after incubation with 0.5 mg/mL osthole histamine-induced secretion of IL-8 was reduced to the level of control.

TNF-α levels were increased after incubation with histamine and LPS, while osthole successively reduced this effect of inflammatory mediators in a concentration-dependent manner. At a concentration of 0.5 mg/mL, osthole showed a better antihistamine effect than CP, as TNF-α levels were significantly lower after incubation with osthole (Figure 5G,H). No significant differences were observed when the 3D skin model was incubated with LPS and osthole or CP. Importantly, in normal conditions osthole at the concentration of 0.5 mg/mL significantly reduced TNF-α secretion compared with the control group (Figure S1L).

**Figure 6:**
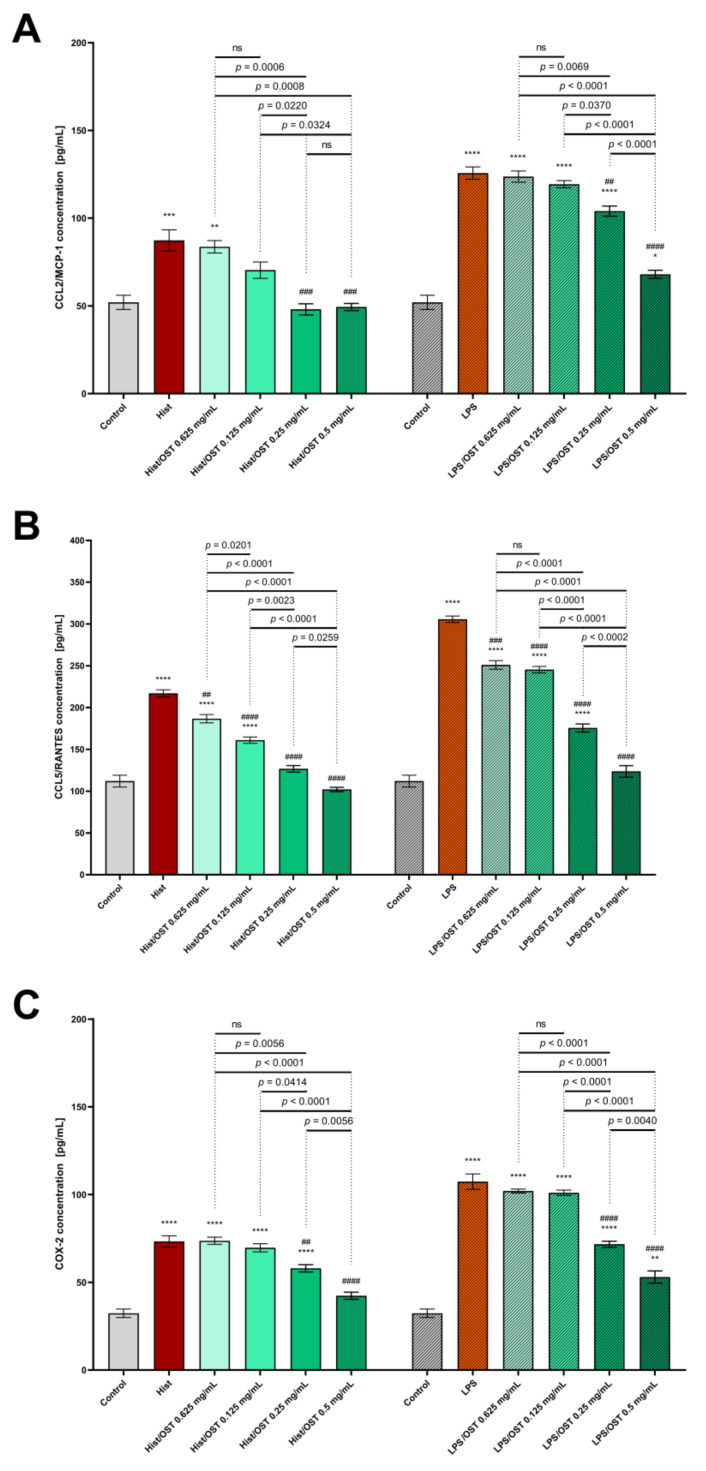
Secretion of CCL2/MCP-1 (**A**); CCL5/RANTES (**B**) and COX-2 (**C**) in the 3D skin model after incubation with histamine (Hist; 100 µg/mL), lipopolysaccharides (LPS; 2 µg/mL) alone and in mixtures with osthole (OST; 0.0625–0.5 mg/mL). The horizontal line shows the mean and the bars show the standard error of the mean. Statistically significant differences (one-way ANOVA with Tukey’s multiple comparisons test) compared to control (*—*p* < 0.05*,* **—*p* < 0.01, ***—*p* < 0.001, ****—*p* < 0.0001) and to cells treated with histamine or LPS (##—*p* < 0.01, ###—*p* < 0.001, ####—*p* < 0.0001) are marked; ns—non-significant.

It was investigated whether osthole affects the secretion of the abundant inflammatory proteins CCL2/MCP-1, CCL5/RANTES and COX-2. After incubation with histamine and LPS, the levels of all studied proteins were increased (Figure 6). Differential efficacy of osthole concentration in attenuating protein secretion induced by histamine or LPS was observed: osthole at concentrations of 0.25–0.5 mg/mL significantly decreased CCL2/MCP-1 levels after incubation with histamine and reached control levels, whereas after incubation with LPS, the two highest concentrations of osthole significantly reduced CCL2/MCP-1 levels (Figure 6A).

The CCL5/RANTES level after incubation with histamine or LPS was reduced by treatment with osthole, and even the lowest concentration of osthole (0.0625 mg/mL) was effective (Figure 6B).

COX-2 levels after histamine/LPS stimulation were significantly decreased when 0.25 and 0.5 mg/mL osthole were administered (Figure 6C). Importantly, osthole decreased the secretion of all tested proteins in a concentration-dependent manner.

### 3.3. Osthole Regulates the Expression of TLR2, TIRAP, MyD88, IRAK1, TRAF6, IκBα and NFκB in Histamine/LPS-Induced Organotypic 3D Skin Model

To investigate the regulatory mechanisms underlying the anti-inflammatory effect of osthole, the expression of *COX-2* and genes involved in TLR2 signaling was studied.

**Figure 7:**
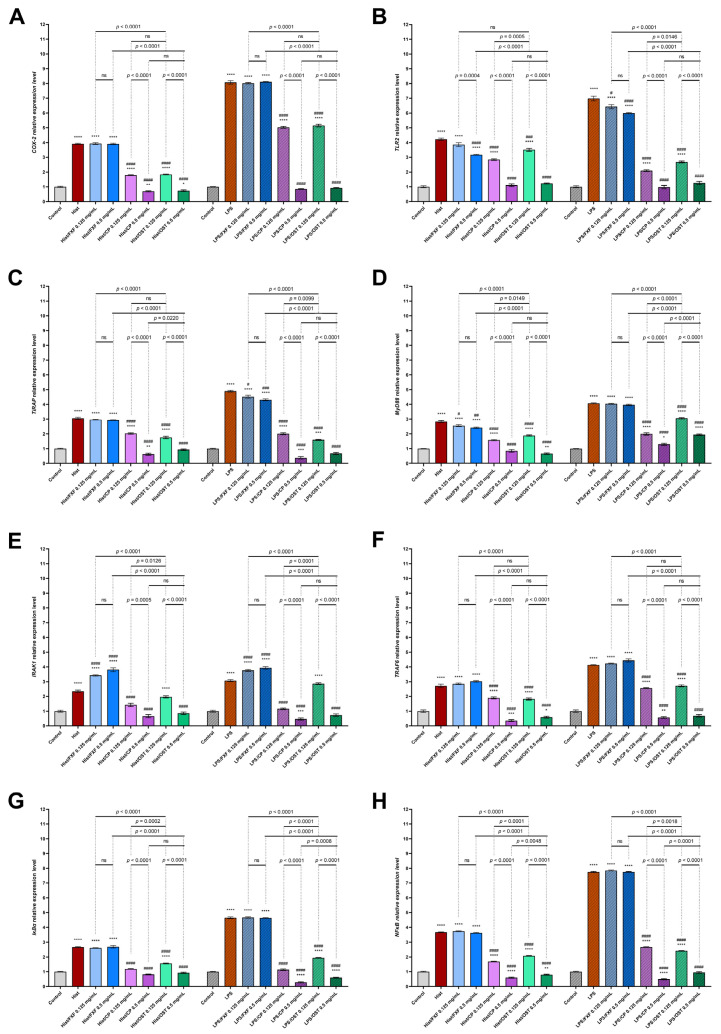
The expression of *COX-*2 and genes involved in TLR2 signaling pathway in 3D organotypic skin model. Expression of *COX-2* (**A**); *TLR2* (**B**); *TIRAP* (**C**); *MyD88* (**D**); *IRAK1* (**E**); *TRAF6* (**F**); *IκBα* (**G**) and *NFκB* (**H**) after incubation with histamine (Hist; 100 µg/mL) or lipopolysaccharides (LPS 2 µg/mL) alone and in mixtures with fexofenadine (FXF) clobetasol propionate (CP) and osthole (0.125 and 0.5 mg/mL) are shown. The horizontal line shows the mean and the bars show the standard error of the mean. Statistically significant differences (two-way ANOVA followed by Tukey’s multiple comparison test) compared to control (*—*p* < 0.05, **—*p* < 0.01, ***—*p* < 0.001, ****—*p* < 0.0001) and to cells treated with histamine or LPS (#—*p* < 0.05, ##—*p* < 0.01, ###—*p* < 0.001, ####—*p* < 0.0001) are marked; ns—non-significant.

Histamine and LPS significantly increased the expression of *COX-2* (Figure 7A), *TLR2* (Figure 7B), *TIRAP* (Figure 7C), *MyD88* (Figure 7D), *IRAK1* (Figure 7E), *TRAF6* (Figure 7F), *IκBα* (Figure 7G) and *NFκB* (Figure 7H) in the 3D skin model.

**Figure 8:**
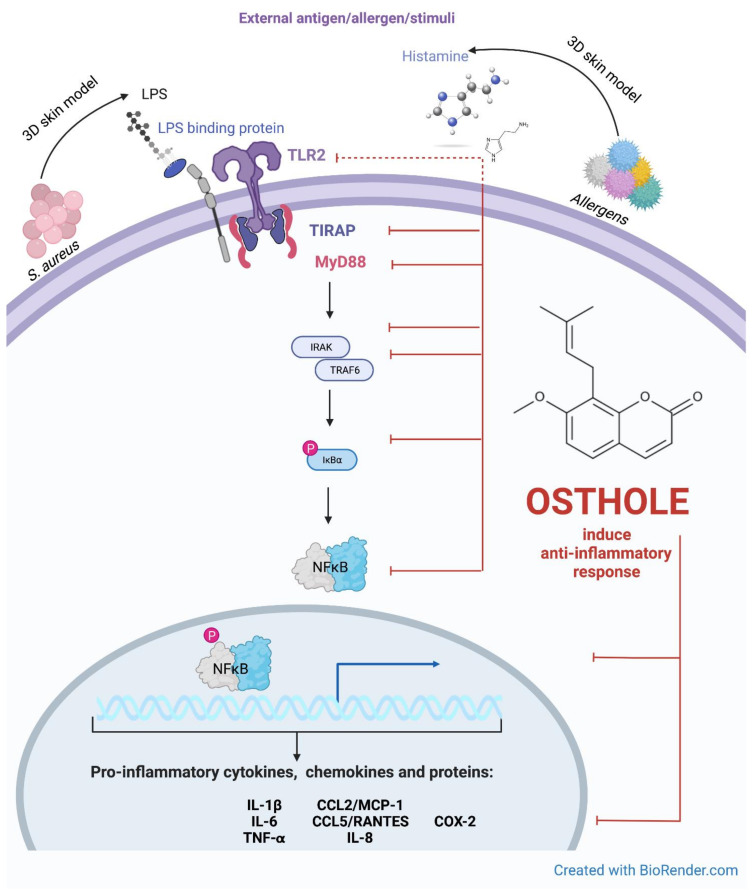
Putative pathway for the inhibitory effect of osthole on the TLR-2 signaling pathway. The interplay between TLR2-mediated signaling and induction of expression of secreted cytokines, chemokines and proteins during stimulation with histamine and LPS in 3D skin model. Created with BioRender.com.

Osthole at a concentration of 0.5 mg/mL significantly decreased the expression of the analyzed genes after incubation with inflammatory mediators. Notably, incubation with osthole at a concentration of 0.5 mg/mL resulted in a decrease in gene expression to the level of control or below (except MyD88 after stimulation with LPS). A similar trend was observed for CP at this concentration. The levels of all genes tested were downregulated by osthole and CP in a concentration-dependent manner. Overall, these results suggest that osthole suppresses histamine- and LPS-triggered activation of pro-inflammatory CKs and ChKs secretion, as well as the expression of genes associated with TLR2 signaling (Figure 8).

## 4. Discussion

AD is a chronic inflammatory skin disease caused by complex genetic, epigenetic, environmental and immunological interactions with an overlapping epidermal barrier defect [42]. Treatment of AD is based on topical therapy with emollients, systemic therapy with anti-inflammatory drugs, antihistamines and antibiotics [43], but most patients take a combination of drugs [44]. Topical steroids are the first choice in the treatment of AD; however, their use is associated with side effects and can lead to steroid phobia [45]. Therefore, natural substances with low side effects have attracted much interest in recent years for the treatment of AD. Natural substances are potential therapeutics for the treatment of AD-related diseases, although scientific evidence for their beneficial effects is insufficient [45,46]. In this study, we investigated the effects of osthole, a natural compound, on the human model of epidermis in induced AD.

In our study, the integrity of the skin barrier was examined by measuring the TEER value. Several studies have shown that TEER allows characterization of intercellular contacts [47] or skin irritation [48]. We have shown that the induction of inflammation in a 3D skin model leads to a decrease in TEER value, providing a tool to study the protective effects of therapeutics. The results show that osthole and CP prevent the loss of cell–cell interactions in the epidermis in a concentration-dependent manner, with osthole being more efficient. Furthermore, stimulation with osthole increased TEER compared to control, indicating an improvement in the integrity of the functioning barrier. Trautmann et al. postulated that keratinocyte apoptosis is a useful parameter to evaluate the activity of eczematous dermatitis [49]. Therefore, monitoring the integrity of the epidermal barrier may provide an indication of lesion severity or treatment efficacy.

Our results clearly demonstrate that administration of osthole can alleviate inflammatory manifestations in an AD-induced skin model. The results showed that osthole reduced the expression of TLR2 and TLR2 signaling-associated genes in vitro. We observed a significant reduction in the secretion of pro-inflammatory CKs on both the basolateral and apical sides of the inserts.

Immunological dysregulation is an important factor in the pathogenesis of AD [50]. Macrophages and PBMCs from AD patients treated with TLR2 ligands secrete less IL-6, IL-1β, IFN-γ, IL-12 and IL-22, but more IL-5 [51,52] TLR2 is normally expressed throughout the epidermis but is restricted to basal keratinocytes in AD [14]. While TLR2 signaling is impaired in the acute phase of AD, it has also been suggested that aberrant activation of TLR2 may play a role in the development of the Th1 immune pathway leading to the exacerbation and persistence of inflammation in the chronic phase of AD [53,54].

The TIR domain is the most important for activating the cascade associated with TIRAP, TRIF, TRAM and MyD88. TLRs selectively target them. Except for TLR3, all signaling pathways require the MyD88 protein. The TIRAP adaptor protein is required for TLR2 activation. This is due to the presence of the identical electrical charges of the TLR2 receptor of the MyD88 adaptor molecule. The surface of MyD88 TLR2 is electropositive, making it impossible for MyD88 to bind directly to this receptor. The TIRAP molecule, on the other hand, has a predominantly electronegative surface that allows the electropositive TLR2 and MyD88 molecules to bind and form a signaling complex. Direct binding of the adapter molecule MyD88 to TLR2 leads to stimulation of the receptor and activation of IRAK-4 kinase. This step, in turn, leads to phosphorylation of IRAK-1. The active IRAK-1 kinase is released into the cytoplasm, where it binds to the TRAF6 factor and activates the TAK1/TAB complex. The stimulated TAK1/TAB complex activates IKK—IκB kinase and MAP—kinase (mitogen-activated protein). Activation of IKK is associated with phosphorylation and degradation of IκBα, leading to the release of transcription factor NFκB. NFκB enters the nucleus and induces the expression of many genes, including those encoding proinflammatory cytokines, adhesive molecules, and numerous enzymes (Figure 1) [55,56,57].

TLR2-4/MyD88/NFκB signaling may facilitate the release of inflammatory cytokines [16,17] Calycosin, a widely used herbal agent in Chinese medicine, reduces allergic inflammation by inhibiting TLR4/MyD88/NFκB [58], indicating a high potential of natural agents in regulating inflammation-associated signaling pathways. The compounds used in Chinese medicine are known to significantly improve the disease state, alleviate the disease progression, shorten the time to disease recurrence and improve the quality of life of patients. Therefore, herbal remedies are increasingly recommended in clinical practice due to their remarkable therapeutic effects in numerous diseases [59].

To further elucidate the mechanism underlying the anti-inflammatory effects of osthole, we investigated its influence on the expression of TLR2 and genes-encoding proteins involved in TLR2 signaling. Impaired TLR2 function has been associated with pathogenesis of AD [60,61]. TLRs are important molecules of innate immunity that recognize conserved structures in various pathogens and either promote or inhibit inflammatory and immune responses [62]. The role of TLRs in the pathophysiology of AD is not fully understood [63]. In addition, polymorphisms in TLR2 are associated with AD, and TLR2 has also been downregulated. In normal keratinocytes, activation of TLR2 leads to a rapid increase in tight junction protein expression in differentiated epidermal layers [61,64]. However, in AD, tight junction protein expression is markedly reduced, suggesting that TLR2 signaling is impaired in the suprabasal layers of the epidermis, where TLR2 signaling-associated proteins are expressed [65,66,67]. We observed lower expression of *TLR2*, *TIRAP*, *MyD88*, *IRAK1*, *TRAF6* in 3D skin models treated with inflammatory mediators and osthole or CP than in skin incubated with histamine and LPS alone. Impaired TLR2 function promotes loss of barrier integrity and immune imbalance in the acute phase of AD [68]. However, abnormal activation of TLR2 may lead to activation of T helper cell-related immune responses and production of keratinocyte-specific CKs and ChKs, which trigger allergic immune responses in the chronic phase of AD [69]. Therefore, strategies that finely modulate TLR2 expression or function promise to restore barrier function and immune balance in AD [64,65,70]. Thus, TIRAP is an essential player in the development of inflammation, which could be successively reduced by stimulation with osthole in a concentration-dependent manner. Over the past decade, numerous clinical and experimental studies have focused on the role of TIRAP in the control of human disease [71,72,73].

In our research, osthole successively reduces *TRAF6* expression in 3D skin models incubated with histamine or LPS. Deficiency of TRAF6 in keratinocytes causes IL-17 psoriatic inflammation in skin [74]. Keratinocyte-specific TRAF6-deficient mice (Traf6EKO mice) show resistance to skin inflammation in AD. In addition, Traf6EKO mice show lower infiltration of immune cells into imiquimod-induced skin inflammation on ears, and lower levels of pro-inflammatory CKs(IL-17 and IL-23) in the place of inflammation [74,75]. TRAF6 signaling in keratinocytes contributes to IL-23 production and activation of skin immunity. TRAF6 is utilized by both TLRs and IL-17 receptors, and study performed by Matsumoto et al. suggests that orchestration of TRAF6 signaling *via* these receptors plays an important role in the initiation and propagation of IL-17-mediated immunity in skin [74]. These signaling pathways are important targets for therapeutic intervention [76,77,78]. In addition, NFκB signaling is known to be an important molecular pathway for TLRs ligand-induced inflammation [75,79]. Activation of TLRs also activates the MAPK signaling pathway, which is involved in many cellular functions [80,81,82,83]. The present study showed that osthole inhibited the expression of IκBα and NFκB induced by histamine or LPS in a 3D skin model. A recent study identified a specific polymorphism of TLR2-5 and -9 in patients with atopic eczema [84,85,86]. To alter TLRs in clinical practice, receptor antagonists, receptor agonists, or single transduction inhibitors can be used [87]. In the present study, we aimed to investigate how TLR2 is related to inflammatory skin diseases and whether it can be used as a therapeutic target. In addition to the complex interactions between impaired epithelial barrier function, receptor expression, signaling pathways and altered cytokine production, TLR-mediated activation or dysfunction has been linked to the development and exacerbation of AD [83]. Wang et al. show that mRNA expression studies are necessary to determine the functional and diagnostic role of the tested drugs in modulating the AD relapse model by regulating gene expression at numerous levels [88]. In the present study, we used osthole to verify its inhibitory effect on TLR2 expression, which is considered a promising therapeutic target to suppress disease-related gene expression.

Pro-inflammatory CKs produced by Th2 lymphocytes, including IL-1β, IL-6 and TNF-α, ChKs such as IL-8, CCL5/RANTES, CC2/MCP-1 and protein-like COX-2, play a central role in the pathogenesis of AD [89]. In this study, treatment with osthole normalized histamine/LPS-mediated immunological dysregulation in AD in 3D skin model by significantly decreasing the secretion of IL-1β, IL-6, IL-8, TNF-α, CCL5/RANTES, CC2/MCP-1 and COX-2.

COX-2 have been associated with severe allergic reactions in AD [90]. Its expression is enhanced in inflammation-related cells in response to stimulations by CKs [91]. Literature indicates that COX-2 [92] and NFκB [93] play important roles in the progression of inflammation by integration and coordination innate and adaptive signals required for the formation of productive immune responses [94]. Thus, the effect of osthole on the expression of COX-2 was determined. Osthole caused a significant decrease in the COX-2 on mRNA (Figure 7A) and protein level (Figure 6C) in histamine- or LPS induced inflammation. We suspect that osthole may be able to inhibit the release of COX-2 *via* inhibiting the translocation of NFκB.

Despite significant advances in the treatment of AD, the long-term therapeutic effects and side effects are not well known due to the various causes of AD [95]. Simultaneous inhibition of other critical genes in AD is expected to achieve remarkable therapeutic effects [96]. The present study has shown that the modulatory effect of osthole may play a role in the therapy of AD if an appropriate concentration of the drug is selected to achieve the maximum therapeutic effect.

## 5. Conclusions

Our study demonstrated the anti-inflammatory effect of osthole in the 3D organotypic skin model through TLR2 *via* inhibition of cytoplasmic adaptor proteins and NFκB expression. Osthole is a promising alternative therapeutic agent for the treatment of allergic and inflammatory diseases, including AD. In the current study, we found that osthole has similar properties in inhibiting immune-mediated skin diseases to CP, which is widely used in the treatment of AD. In addition, we found that osthole regulates the expression of TLR2 and genes-encoding cytoplasmic adaptor proteins, which is critical in the immunomodulation of AD. We suppose that the concomitant use of other drugs with osthole could improve the therapeutic outcomes of AD therapy and enhance its clinical application in the future.

In view of the present results, the development and validation of new approaches for the treatment of AD is recommended, such as the possible use of antagonists/inhibitors of the TLR pathway to preferentially limit the association of TLRs with MyD88 and reduce NFκB activity and/or CKs and ChKs secretion.

## Data Availability

The data presented in this study are available on request from the corresponding author.

## Supplementary Materials

The following are available online at https://www.mdpi.com/article/10.3390/cells11010088/s1: Table S1. Sequences of the oligonucleotide primers specific to examined genes; Figure S1. The levels of IL-1β (A–C), IL-6 (D–F), IL-8 (G–I) and TNF-α (J–L) in the 3D skin model after incubation with fexofenadine (FXF), clobetasol propionate (CP), and osthole (OST). The horizontal line shows the mean and the bars show the standard error of the mean. Statistically significant differences (one-way ANOVA with Tukey’s multiple comparisons test) compared to control (*—*p* < 0.05, **—*p* < 0.01,***—*p* < 0.001, *p* < 0.0001) are marked; ns—non-significant.

## Abbreviations

AD	Atopic dermatitis
CCL2/MCP-1	Chemokine (C-C motif) ligand 2/monocyte chemoattractant protein 1
CCL5/RANTES	Chemokine (C-C motif) ligand 5/(regulated on activation, normal T cell expressed and secreted
ChKs	Chemokines
CKs	Cytokines
COX-2	Cyclooxygenase 2
CP	Clobetasol propionate
DMSO	Dimethyl sulfoxide
ELISA	Enzyme-linked immunosorbent assay
FXF	Fexofenadine hydrochloride
IFN	Interferon
IRAK1	Interleukin receptor-associated kinase 1
IκB⍺	NFκB inhibitor alpha
LPS	Lipopolysaccharides
MyD88	Myeloid differentiation protein 88
NFκB	Nuclear factor kappa B
NHDF	Normal human dermal fibroblasts
NHEK	Normal human epidermal keratinocytes
PBMCs	Peripheral blood mononuclear cells
TEER	Transepithelial electrical resistance
TIRAP	TIR domain containing adaptor protein
TLR2	Toll-like receptor 2
TNF-α	Tumor necrosis factor alpha
TRAF6	TNF receptor-associated factor 6
