# Supplementary material for: Osthole Inhibits Expression of Genes Associated with Toll-like Receptor 2 Signaling Pathway in an Organotypic 3D Skin Model of Human Epidermis with Atopic Dermatitis
Source: Cells. 2021 Dec 28;11(1):88. doi: 10.3390/cells11010088 (PMC8750192; doi:10.3390/cells11010088)
Supplement: Supplementary file 1 [file cells-11-00088-s001.zip › Supplementary files/Table S1.pdf]

**Table S1.** Sequences of the oligonucleotide primers specific to examined genes.

| Gene | Gene ID | Sequence | Product length (bp) |
|---|---|---|---|
| *YWHAZ* | 7534 | F: 5'- TGTAGGAGCCCGTAGGTCATC -3'<br>R: 5'- GTGAAGCATTGGGGATCAAGA -3' | 179 |
| *TLR2* | 7097 | F: 5'- AGTTTCCAACACCCCTCCTG -3'<br>R: 5'- CATTGTCCAGTGCTTCAACCTTT -3' | 237 |
| *NFκB* | M58603.1 | F: 5'-TGGAGTCTGGGAAGGATTTG-3'<br>R: 5'-CGAAGCTGGACAAACACAGA-3' | 129 |
| *TIRAP/Mal* | 114609 | F: 5'-GCTGTCATGCGTTATCTGC-3'<br>R: 5'-CACATTTGTGGGAATCCGAGG-3' | 118 |
| *MyD88* | 4615 | F: 5'- CCTTCATCTGCTATTGCCCCAG -3'<br>R: 5'- CCTTCTAGCCAACCTCTTTTCG -3' | 170 |
| *IRAK* | 3654 | F: 5'- CTACAAGAAGCACCTGGACCC -3'<br>R: 5'- TCTAGCCTCTCGTACACCTGGG -3' | 138 |
| IKB-*α* | 4793 | F: 5'- CCTGGTGTCACTCCTGTTGA -3'<br>R: 5'- AGTCATCATAGGGCAGCTCGTC -3' | 230 |
| *TRAF6* | 7189 | F: 5'- TGGCAGACTGTGACACTCAA -3'<br>R: 5'- TGACCCGAATTTCCTGTGGG -3' | 140 |
